# *De Novo* Transcriptome of the Hemimetabolous German Cockroach (*Blattella germanica*)

**DOI:** 10.1371/journal.pone.0106932

**Published:** 2014-09-29

**Authors:** Xiaojie Zhou, Kun Qian, Ying Tong, Junwei Jerry Zhu, Xinghui Qiu, Xiaopeng Zeng

**Affiliations:** 1 Institute of Disinfection and Vector Control, Beijing Center for Disease Control and Prevention, Beijing, China; 2 State Key Laboratory of Integrated Management of Pest Insects and Rodents, Institute of Zoology, Chinese Academy of Sciences, Beijing, China; 3 United States Department of Agriculture, Agricultural Research Service, Lincoln, Nebraska, United States of America; Instituto Oswaldo Cruz, Fiocruz, Brazil

## Abstract

**Background:**

The German cockroach, *Blattella germanica*, is an important insect pest that transmits various pathogens mechanically and causes severe allergic diseases. This insect has long served as a model system for studies of insect biology, physiology and ecology. However, the lack of genome or transcriptome information heavily hinder our further understanding about the German cockroach in every aspect at a molecular level and on a genome-wide scale. To explore the transcriptome and identify unique sequences of interest, we subjected the *B. germanica* transcriptome to massively parallel pyrosequencing and generated the first reference transcriptome for *B. germanica*.

**Methodology/Principal Findings:**

A total of 1,365,609 raw reads with an average length of 529 bp were generated via pyrosequencing the mixed cDNA library from different life stages of German cockroach including maturing oothecae, nymphs, adult females and males. The raw reads were *de novo* assembled to 48,800 contigs and 3,961 singletons with high-quality unique sequences. These sequences were annotated and classified functionally in terms of BLAST, GO and KEGG, and the genes putatively coding detoxification enzyme systems, insecticide targets, key components in systematic RNA interference, immunity and chemoreception pathways were identified. A total of 3,601 SSRs (Simple Sequence Repeats) loci were also predicted.

**Conclusions/Significance:**

The whole transcriptome pyrosequencing data from this study provides a usable genetic resource for future identification of potential functional genes involved in various biological processes.

## Introduction

Cockroaches are one of the most ancient and primitive winged insects, which have existed successfully and remained virtually unchanged in body morphology for approximately 350 million years from the Carboniferous to present [1]. There are about 3,500 known species of cockroaches globally, only thirty are considered as household pests. Among them, the German cockroach (*Blattella germanica*) is listed as one of the most important public health related insect pests [2], because it is highly dependent on human for survival and can transmit mechanically a number of pathogenic viruses, fungi, helminths and bacteria (with some exhibiting resistance to antibiotics) [3], [4]. For nearly a half century, the worldwide occurrence of allergic respiratory morbidity, especially in childhood, has been considered to be closely related to the German cockroach infestation [5], [6]. On the other hand, the German cockroach, a phylogenetically basal species showing a gradual metamorphosis, has also been considered as an important model for scientific studies regarding reproduction, metamorphosis, neurophysiology, behavioral and chemical ecology, allergen biology, nutrition metabolism and insecticide resistance [1].

Despite its medical significance and prominence as a model in entomological study, our understanding about the German cockroach has been largely hindered by the lack of thorough genetic information. Previous efforts in this direction were limited to a few low-throughput EST projects such as subtractive hybridization [7] and cDNA library Sanger sequencing [8]. Until now, only 2,876 short ESTs are publicly available at the NCBI (National Center for Biotechnology Information) website. No complete picture has been achieved even at the scale of a specific gene family. For example, cytochrome P450s (CYPs) have been highly recognized as a super gene family with 36 to 180 genes existed in insect genomes [9], while only 7 CYP genes with a full-length coding region can be accessed for *B. germanica* in GenBank. This scarcity of genetic information in *B. germanica* has also resulted in a paucity of genetic studies and integrated theories for understanding its basic biology.

*B. germanica* was in the list of the 5,000 Insect Genome Project (i5k), a huge project that has been initiated recently. EST or transcriptome sequencing could not only be useful for the assembly and annotation of genomic data, but also as an efficient and feasible alternative approach to obtain genetic information. Next-generation sequencing (NGS) platforms, such as Roche 454 Genome Sequencer FLX System (454), Illumina Genome Analyzer (Solexa) and Applied Biosystems SOLiD system (SOLiD), have been increasingly used and dramatically accelerated biological and biomedical research on a genome-wide scale. The most significant advantage of NGS over traditional Sanger-sequencing is its massively parallel sequencing ability with significant low cost for DNA sequencing [10]–[12]. The 454 pyrosequencing is very suitable for a non-model species, enabling high efficient *de novo* sequencing, assembly and annotation of expressed genes [13], [14], thus has been widely applied in a broad range of arthropod species including *Cimex lectularius* [15], [16], *Anopheles funestus* [17], *Aedes aegypti* [18], *Dermacentor variabilis* [19], *Musca domestica* [20], *Sarcophaga crassipalpis* [21], *Cochliomyia hominivorax* [22], *Anastrepha suspensa* [23], *Nilaparvata lugens* [24],*Manduca sexta* [25], *Lutzomyia intermedia* [26], *Amblyomma maculatum* [27], *Corethrella appendiculata* [28] and *Rhodnius prolixus* [29]. For the German cockroach, Illumina sequencing was used to identify microRNAs from the ovaries and whole body of 6^th^ instar nymphs [30]. In the present study, we attempt to explore the transcriptome of *B. germanica* using 454 pyrosequencing. We hope that the generated transcriptome database can serve as a valuable resource for better understandings of the molecular mechanisms underlying important biological processes and the environmental adaptability of the cockroach.

## Materials and Methods

### Ethics Statement

The German cockroach used in the present study is a common indoor insect pest, not an endangered and protected species. No permission was required to sample and collect the German cockroaches from the infested restaurants, where we scheduled routine cockroach density surveillance plan for the public health purpose.

### Cockroaches

Two strains of German cockroach (*B. germanica*) BJ-S and DX-R, were used in this study. Both strains were kept separately in glass jars of the same size (13 liters), coated with petroleum jelly (top 1/5 of the jar) at 26±1°C and 60±10% relative humidity with a photoperiod of 12∶12 (L:D) h. Cockroaches were fed with laboratory rodent food (Beijing Huafukang Biotechnology) and water *ad libitum*. The susceptible strain (BJ-S) had been reared in the laboratory without exposure to any insecticide since 1970. A field strain (DX-R) was established from cockroaches collected in a local restaurant (Daxing district, Beijing) in 2011. The comparison in resistance of the two strains to several types of insecticides is shown in Table S1. The inclusion of this field collection in this study was to increase the genetic diversity and to obtain preliminary information about the differentially expressed genes associated with insecticide resistance for future studies. Cockroach samples for RNA extraction and quantification were collected and snap-frozen immediately in liquid N_2_ and stored at −80°C until use.

### RNA isolation, cDNA library construction and 454 pyrosequencing

In order to obtain a transcriptomic dataset with a coverage as wide as possible, RNA was extracted from pooled samples of 30 maturing oothecae, 30 4^th^ instar nymphs, 30 adult females and 30 adult males (7 days after eclosion) respectively from each strain. Total RNA was extracted using the Trizol reagent (Invitrogen, CA, USA) according to the manufacturer's instruction. The integrity of total RNA was assessed by both 1.4% denaturing formaldehyde agarose gel electrophoresis and the Agilent 2100 Bioanalyzer (Palo Alto, CA, USA) with a minimum integrity value of 8. The quantity of total RNA was determined by NanoDrop 1000 spectrophotometer (Thermo, MA, USA). Equal quantities (10 µL) of total RNA (1 µg/µL) from each of the four life stages were combined to form a RNA pool for each strain. mRNA was isolated by using PolyATtract mRNA isolation systems (Promega, WI, USA) from each total RNA pool. mRNA pools were concentrated by using RNeasy MinElute Cleanup Kit (Qiagen, Valencia, CA) and used as starting material for cDNA library construction.

Two cDNA libraries of BJ-S and DX-R strains were separately constructed from respective pooled mRNA samples for 454 pyrosequencing. Briefly, the mRNA was broken into short fragments in the presence of fragmentation buffer at 94°C for 5 min. These short fragments were used as templates for first-stranded cDNA synthesis using random hexamer primers. Subsequently, second-stranded cDNAs were synthesized using dNTPs, RNaseH and DNA polymerase I. DNA bands (500–800 bp) were excised and purified from agarose gels using the QIAquick Gel Extraction Kit (Qiagen, Valencia, CA). The isolated double-stranded cDNA were blunt-ended using T4 DNA polymerase and T4 polynucleotide kinase (PNK), then ligated to the adapters (Titanium A&B provided in the 454 library kit) with T4 DNA ligase, followed by immobilization on DNA capture beads. DNA capture beads were clonally amplified by emulsion PCR (emPCR) and enriched by removing the waste oil from the beads and selecting for beads with amplified library fragments. The beads were counted using a Beckman Coulter Z1 Particle Counter and loaded into the wells of PicoTiter plate (PTP). A single full PTP was sequenced with a half plate for each library following standard protocols described by Margulies [12] on a 454 GS FLX Titanium instrument (Roche Diagnostics, Indianapolis, IN).

### Sequence preprocessing and *de novo* assembly

The raw 454 reads were produced by the base-calling process which transformed the measured pyroluminescence intensity signals to a sequence of nucleotides. These read can be accessed through NCBI Short Read Archive (SRA) under the accession code SRP042142. Raw reads were preprocessed by in-house developed tools, TagDust [31] and Seqclean [32] to trim the adapter, poly A/T tails and remove low-quality, short read data and contamination sequences. The resultant clean reads from both strains respectively were combined to assemble into unique sequences by the Newbler [12] assembler programs at default parameters. This Transcriptome Shotgun Assembly project has been deposited at DDBJ/EMBL/GenBank under the accession GBID00000000. The version described in this paper is the first version, GBID01000000.

### Homology searches and EST annotation

The generated unigenes were firstly BLASTx searched against the Swiss-Prot [33] and NCBI non-redundant protein database. The sequences retrieving no BLASTx hit were searched by BLASTn against the NCBI nucleotide collection. Gene ontology (GO), KOG [34] and KEGG [35] analysis were used for functional classification of the annotated ESTs. For gene ontology analysis, both BLAST2GO [36], [37] and WEGO [38] were employed. The programs extracted the GO terms associated with homologies identified with BLAST and returned a list of GO annotations represented as hierarchical categories of increasing specificity.

### Identification and analysis of genes of interest

Genes of interest were further manually examined to check for possible frameshifts caused by the 454 sequencing based on the annotated ESTs. All of the manually confirmed protein sequences were used for alignment and phylogenetic analysis. Alignments were implemented using BioEdit, and employed to reconstruct the phylogeny by using the MEGA6 software [39]. The neighbor-joining method was used to create phylogenetic trees with p-distance under the default parameters of MEGA program. Bootstrap analysis of 1000 replications was performed to evaluate the branch strength of each tree.

## Results and Discussion

### Pyrosequencing, assembly and annotation

**Figure 1:**
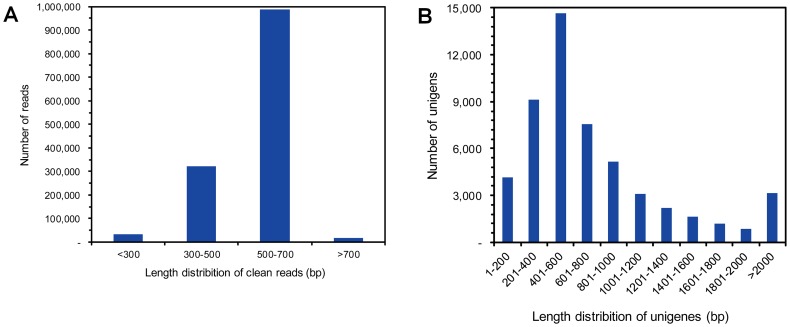
Length distribution of clean reads (A) produced by 454 pyrosequencing and the assembled unigenes (B).

Using Roche 454 GS-FLX platform, our single full run from two cockroach strains with each strain half-run yielded a total of 1,365,609 raw reads with an average length of 529 bp. After preprocessing the raw data (including adaptor trimming and removal of low-quality reads), 1,362,260 reads remained with a total amount of 42,110,570 bp and an average length of 520 bp. All the clean reads were assembled into 48,800 contigs and 3,961 singletons forming a total of 52,761 high-quality unigenes with an average length of 798 bp (Table 1). Of these unigenes, 12,146 (23.0%) were over 1000 bp in length, and 5504 (36.7%) were between 500–1000 bp. The length distribution of the reads and unigenes is shown in Figure 1.

**Table 1:** Summary statistics of the ESTs generated from the German cockroach through pyrosequencing.

Features	Values
Total number of reads	1,365,609
Average read length	529 bp
Number of reads involved in the assembly	1,362,260
Total number of unique sequences	52,761
Total number of contigs	48,800
Total number of singletons	3,961
Average unigenes size	798 bp
N50 contig size	792 bp

Through the BLASTx-BLASTn sequential homology search, nearly half (47.7%) of the unigenes could be annotated. When all unique transcripts were aligned against the Swiss-Prot database using BLASTx, a total of 17,779 (33.7%) unique transcripts yielded one or more significant hits. Table 2 lists the characteristics of the top 20 most abundant ESTs (the sequences are in File S1). Besides some housekeeping high-abundant genes, some genes involved in the reproduction (vitellogenin), defence (transferrin), energy metabolism (COX1), detoxification enzyme (CYP4G19) and allergenic proteins also were expressed at high levels. The number of identified genes related to some important physiological functions in the German cockroach is shown in Table 3.

**Table 2:** Summary of top 20 abundant assembled unigenes from the German cockroach transcriptome.

Unigene ID	Length (bp)	Top Blast hit	Identity (%)
Contig6820	4019	18S ribosomal RNA, *Blattella germanica*	99
Contig4068	6792	Vitellogenin-2, *B. germanica*	99
unigene_rep_c6381	887	NADH dehydrogenase subunit 1, *B. germanica*	100
Contig3091	2293	Transferrin, *Blaberus discoidalis*	82
Contig3072	4114	Myosin heavy chain, *Musca domestica*	91
Contig6443	1518	Cytochrome c oxidase subunit 1 (COX1), *B. germanica*	96
Contig3066	1053	Arginine kinase, *B. germanica*	99
Contig3115	7089	Apolipophorins, *Locusta migratoria*	47
Contig2345	1323	Elongation factor 1α (EF-1α), *Cryptocercus punctulatus*	98
Contig1101	1446	α-Amylase, *B. germanica*	76
Contig946	746	Tropomyosin-2, *Coptotermes formosanus*	96
Contig61	1120	β-actin, *Acyrthosiphon pisum*	100
Contig4872	2616	Elongation factor 2 (EF-2), *Schistocerca gregaria*	99
Contig594	483	Allergen Bla g 8, *B. germanica*	100
Contig848	996	Troponin T (TnT), *C. formosanus*	93
Contig1104	1608	Pyruvate kinase, *Tribolium castaneum*	80
Contig3092	1032	Aspartic protease Bla g 2, *B. germanica*	80
Contig611	1635	Cytochrome P450 CYP4G19, *B. germanica*	100
Contig3123	715	ATP-dependent RNA helicase p62, *T. castaneum*	85
Contig3084	2631	Paramyosin, *Megachile rotundata*	84

**Table 3:** Genes related to important physiological functions.

Functional genes	No. of unigenes	Functional genes	No. of unigenes
**Detoxification**		α-amylase	46
Cytochrome P450	163	α-glucosidase (maltase)	5
GSTs	64	β-glucosidase	21
CarE	12	Lipase	41
ATP-binding cassette	19	**Immunity and defence**	
UDP glycosyltransferases	5	β-glucanase	18
**Insecticide targets**		Lectin-like protein	115
Acetylcholinesterase	3	**Peritrophic membrane biosynthesis and metabolization**	
GABA receptor	2	Chitinase	37
Nicotinic acetylcholine receptor	5	Chitin synthase	5
Sodium channel	6	Chitin deacetylase	3
Glutamate receptor	12	Peritrophin (mucin)-like	19
**Digestion**		**Olfactory reception**	
Serine proteinase all types	19	Odorant-binding protein (OBP)	15
Cysteine proteinase all types	10	Chemosensory protein (CSP)	12
Carboxypeptidase all types	53	Odorant receptor (OR)	2
Aminopeptidase all types	47	**Gustatory reception**	
Dipeptidyl-peptidase	2	Gustatory receptor (GR)	4

### Functional classifications of annotated ESTs

**Figure 2:**
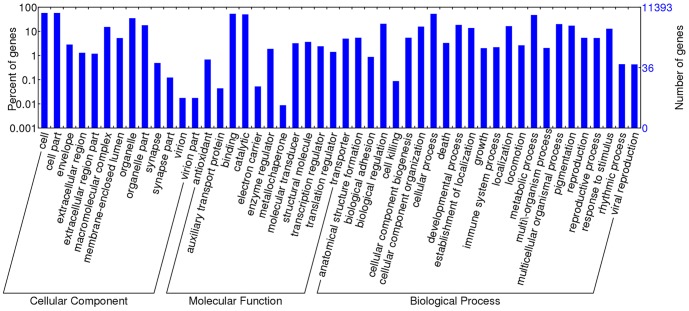
Gene ontology (GO) terms for the transcriptomic sequences of *Blattella germanica*.

GO analysis was conducted using BLAST2GO and WEGO programs. Of the 52,761 unigenes, 11,383 could be assigned into 48 functional groups (Figure 2) in three categories. The three categories were further divided into over 100 sub-categories (Figure S1). Among them, cell and cell part in the cellular component, hydrolase and nucleotide binding in the molecular function, cellular processes and metabolic processes in the biological processes represented the major sub-categories. The smallest groups were the metallochaperone in the molecular function category and the virion part in the cellular component. Some unigenes were assigned to multiple categories of GO terms, while others could not be assigned to a given GO term. The biological process terms were associated predominantly with cellular processes such as proteolysis, carbohydrate metabolic processes and oxidation reduction utilization. Similar composition and distribution of unigenes assigned by GO terms have been reported in transcriptomic description from other insects [15], [20], [40], [41].

**Figure 3:**
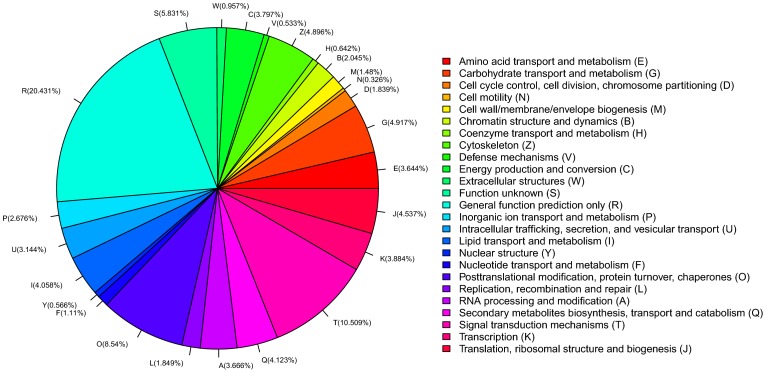
Eukaryotic orthologous groups (KOG) function classification of 52,761 unigenes of *Blattella germanica*.

All unique transcripts were also searched against the KOG database for functional prediction and classification. With some of these unigenes no appropriate KOG annotation, 16,612 KOG annotations were produced and could be classified into 25 molecular families (Figure 3). Among the KOG classifications, the cluster of general function (20.4%) was the largest, followed by translation and signal transduction mechanisms (10.5%), and posttranslational modification, protein turnover, and chaperones (8.5%). The three smallest groups were nuclear structure (0.57%), defense mechanisms (0.53%) and cell motility (0.33%) respectively.

Among all the unigenes, 10,402 sequences with an enzyme classification (EC) number were mapped into 190 KEGG pathways in total (Table S2). Among the pathways identified, the metabolic pathways were found to be the most active, which is similar to the pattern observe *in N. lugens* [24].

### Identification of simple sequence repeats (SSRs) loci

SSRs, also known as microsatellites, are tandem repeated motifs of 1–6 bases and serve as the most important molecular markers in population and conservation genetics, molecular epidemiology and pathology, and gene mapping. By screening all the unigenes, 3601 SSRs loci with di-, tri-, tetra- and penta-nucleotide repeats were identified. Among them, trinucleotide repeats were the most abundant (60.7%), followed by dinucleotide repeats (24.0%), tetranucleotide (13.3%) and pentanucleotide repeats (2.0%). The most frequent motifs were (AAT)n (6.39%) and (TAT)n (5.69%). These SSRs could represent a valuable biomarker resource of *B. germanica*. However, all these putative SSR markers need to be validated to eliminate possible false positives and sequencing errors.

### Transcripts involved in systematic RNA interference pathway

The RNAi process has been fully investigated in *D. melanogaster* and *Caenorhabditis elegans*. Previous studies suggested that systematic RNAi is very efficient in cockroach functional gene study [42]. Diverse proteins such as SID-1 and scavenger receptors (SRs), have been reported to be involved in RNAi process. SID-1 is a protein that transports dsRNA into cells as observed in *C. elegans*; however, no SID-1 orthologue was found in the present transcriptome dataset or other *Dipteran* insects [43]. Over 90% of the dsRNA uptaken into *S2* cells in *Drosophila* is initiated by two SRs, including SR-CI and the scavenger receptor Eater [44]. Gene orthologous to SR-CI was absent herein, and unigene_c62 was identified as Eater, similar to what found in the fruit fly, *Bactrocera dorsalis* [45]. Whether unigene_c62 participates in the uptake of dsRNA in *B. germanica* remains to be investigated.

In general, the dsRNA or short hairpin RNA incorporated into cells is processed into small interfering RNA (siRNA) or microRNA (miRNA) by two distinct Dicer complexes (Dicer-1 and Dicer-2), respectively. Dicer-1 is ATP-independent and prefers to process the stem-loop precursor of miRNA, while Dicer-2 favors long dsRNA as its ideal substrate, and requires ATP hydrolysis for efficient siRNA production [46]. R2D2 can form the Dicer-2/R2D2 complex with Dicer-2, and bind to siRNA to enhance sequence specific messenger RNA degradation mediated by the RNA-initiated silencing complex (RISC). In *Drosophila*, R2D2 acts as a bridge between the initiation and effector steps of the RNAi pathway by facilitating siRNA passage from Dicer to RISC [47]. In the present study, we found four unigenes homologous to two Dicer genes of the German cockroach which were fully sequenced with known function [48], while R2D2 lost in the unigene dataset is probably due to the limitation of sequencing coverage.

The Argonaute 2 (AGO2) has been reported to be another main component of RISC complex involved in cleavage of siRNA-directed mRNA and degradation of the passenger strand in the siRNA duplex [49]. We found two Argonaute genes, AGO1 (unigene_c2208) and AGO2 (unigene_c24957), which show high homology to the counterparts of *T. castaneum* (XM_966202) and *Acyrthosiphon pisum* (XM_001944817). Further molecular cloning and functional analyses of these genes may shed light on their roles in the systemic RNAi pathway in the German cockroach.

### Transcripts encoding detoxification enzymes and insecticide targets

German cockroach has been documented to evolve resistance to insecticide rapidly [50]. The known mechanisms underlying insecticide resistance in the German cockroach include decreased penetration [50], increased detoxification [51], target insensitivity [52] and bait aversion [53]. To screen genes that may evolve insecticide resistance, we mined the current transcriptomic data in order to identify unigenes encoding insecticide targets or detoxification enzymes. As shown in Table 3, a number of sequences homologous to detoxification enzymes including carboxylesterase (CarE), glutathione *S*-transferase (GST), cytochrome P450 and insecticide targets were identified. The mean lengths of these unigenes ranged from 387 bp to 985 bp leading to a relatively reliable annotation.

A total of 163 P450-like transcripts with a size of more than 600 bp were identified and curated manually. This gene number fell within the range of insects that their whole genomes were sequenced [9], covering the 13 P450 ESTs previously deposited in GenBank. Based on the closest BLAST hits in the NCBI nr database, these P450 unigenes were tentatively assigned to appropriate CYP families and clades, containing representatives of all the 4 main insect P450 clans (CYP2, CYP3,CYP4 and mitochondrial). CYP3 ranked as the largest clan, consisting of 45 members belonged to the CYP6 family and 28 genes to CYP9. Members from CYP3 clan appear to share the characteristics of environmental response genes, such as very high diversity and rapid rates of evolution [54]. The CYP4 clan included 39 P450s from the CYP4 family. The remainder belonged to the mitochondrial (15 ones) and CYP2 (22 ones) clan, which might be involved in the ecdysteroid metabolism pathway (CYP301 family) and essential physiological function (CYP303–305 and CYP15 families) respectively. The observation that the members of CYP4, CYP6 and CYP9 families together account for 74.2% of the total P450s indicates that the German cockroach, similar to other omnivorous insects, arms itself with potent capacity of metabolizing various xenobiotics.

A total of 64 GST-related EST sequences were identified in our database. However, only 12 contigs possessing a characteristic motif of CarE were found, which are relatively fewer than those found in other insects. In addition, a number of contigs encoding insecticide target proteins were also identified in the transcriptome, including acetylcholinesterase (AChE) (3 unigenes), nicotinic acetyl choline receptor subunits (nAChRs) (2), gamma-aminobutyric acid (GABA) receptor (2), glutamate receptor (12) and sodium channel (6).

### Transcripts involved in innate immunity

Insects have powerful innate immunity against many pathogens [29]. Cockroaches encounter various types of infectious agents, because they live under diverse unsanitary and unhygienic milieus. It is logical to believe that cockroaches have developed effective innate immunity of protecting themselves against pathogenic microorganisms. Production of diverse antimicrobial peptides (AMPs) plays critical roles in insect immunity [55]. The AMPs are regulated by a balance between two signaling pathways involving the Toll pathway that is activated mainly by fungi and Gram-positive bacteria, and the Immune deficiency (Imd) pathway that is activated mainly by Gram-negative bacteria. Toll and Imd cascades also control the majority of the genes regulated by microbial infection in addition to AMP genes, and are involved in nearly all known *Drosophila* innate immune reactions [56]. Previous studies suggest that the brain lysates, fat body or haemolymph of American cockroaches (*Periplaneta americana*) have remarkable antimicrobial activity against different bacteria including *Staphylococcus aureus*, MRSA (Gram-positive methicillin-resistant *S. aureus*), *S. epidermidis* and a neuropathogenic *Escherichia coli* K1 [55].

Dozens of genes identified in our transcriptome dataset may contribute to immune response in *B. germanica* (Table 4), including GNBP, PGRP (Peptidoglycan recognition proteins), SR-b/c, Toll-like receptors, Imd (Immune deficiency gene), Lipopolysaccharide-binding protein (LPS-bp), Iap (Inhibitor of apoptosis), MAPK (mitogen-activated protein kinase), prophenoloxidase, lysozymes, galectin, cathepsins, transferrins, defensins, diptericins and serpins. Most of them have a counterpart in other insects such as *Aedes aegypti* [18], *Musca domestica* [20] and *Nilaparvata lugens* [24]. These genes have been documented to play important roles as immune effect factors against possible pathogenic bacteria [55]–[57].

**Table 4:** Genes related to innate immunity.

Tentative annotation	Description	No. of unigenes
GNBP	Gram negative bacteria binding protein	4
PGRP	Peptidoglycan recognition protein	15
SR-b/c	Scavenger receptor which participates in the removal of many foreign substances such as gram (+/−) bacteria	6
Toll-like receptor	Key receptor in Toll pathway	16
Imd	Immune deficiency gene encoding death domain-containing protein	3
LPS-bp	Lipopolysaccharide-binding protein	21
Iap	Inhibitor of apoptosis	4
MAPK	Mitogen-activated protein kinase	2
Prophenoloxidase	Activate the phenoloxidase involved in melanization of pathogens and damaged tissues	2
Lysozyme	Break the bonds between polysaccharides in peptidoglycan on the bacterial cell walls	8
Galectin	Function in insect innate immune as a pattern-recognition protein	3
Cathepsin	Participate in molting, tissue remodeling, embryogenesis and immune evasion	32
Transferrin	An iron transporter function as antibiotic agent, vitellogenin, and juvenile hormone regulated protein	20
Defensin	Function as host defense peptide against bacteria, fungi and viruses.	2
Diptericin	A novel anti-bacterial peptide	4
Serpin	Regulate the innate immune pathway such as Toll by inhibition of serine proteases	47

### Transcripts involved in olfactory and gustatory perception

We identified gene families that have been implicated in chemosensory reception (Table 3) including odorant binding proteins (OBPs), chemosensory proteins (CSPs), odorant receptors (ORs) and gustatory receptors (GRs). The detailed EST sequences are present in File S1.

**Figure 4:**
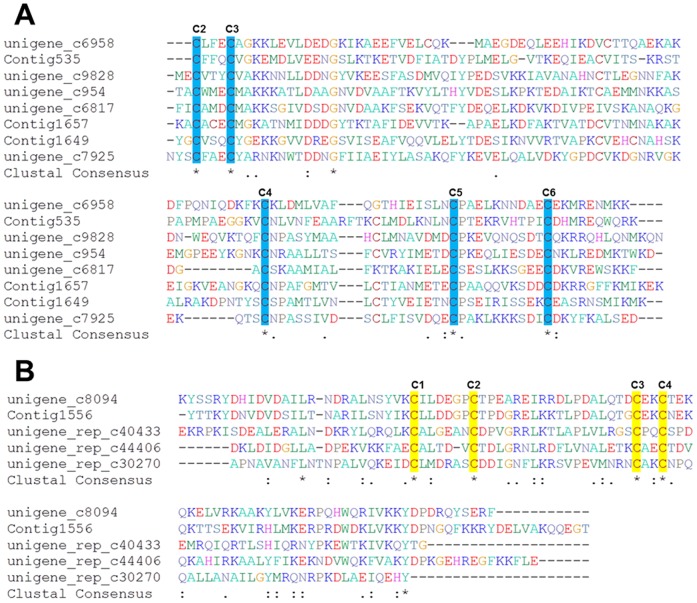
Multiple sequences alignment of deduced peptide sequences of the putative German cockroach OBPs (A) and CSPs (B) with those from other insect species. Nearly full-length amino acid sequences are aligned by ClustalX in BioEdit. Residues shaded in blue and yellow colors show the five and four conserved cysteine residues in the alignment of OBPs and CSPs, respectively. Asterisks (*) indicate identical sites in the sequence alignments; colons (:) represent sites with conserved substitutions, and black dots (•) stand for sites with weakly conserved sites.

OBPs have been proposed to capture and transport hydrophobic chemicals from air to ORs in the lymph of sensory hairs. Fourteen putative OBP transcripts were identified in this study. The number of OBPs identified here was far less than that in *D. melanogaster* (51) [58], *Bombyx mori* (44) [59], *Anopheles gambiae* (69) [60], *Aedes aegypti* (111) [60] and *Culex quinquefasciatus* (109) [60]. We assume that there are still other unidentified OBPs in the German cockroach, although relatively fewer genes encoding OBPs (19 and 5, respectively) were identified in the fire ant *Solenopsis invicta* [61] and the body louse *P. humanus* [62] genomes. An alignment of the deduced German cockroach OBPs (Figure 4A) revealed the most striking six conserved cysteine residues (the first cysteine C1 is absent because of the restriction of assembled unigenes lengths), which are present in the characteristic positions in all known insect OBPs.

CSPs exhibit an expression in the antenna similar to OBPs, and may perform tasks ranging from ontogeny to colony level regulation [63]. CSPs are characterized by only 4 conserved cysteines forming two non-interlocked disulfide bridges, whereas 6 conserved cysteines are paired in three interlocked disulphide bridges in OBPs [64]. Therefore, CSPs may represent a new class of soluble carrier proteins involved in insect chemoreception. The CSP gene family varies in sizes across arthropods. For example, the tick *Ixodes scapularis* has 1 CSP gene, *D. melanogaster* has 4, and larger known repertoires are found in *T. castaneum* (19 genes), *B. mori* (22 genes) and *S. invicta* (21 genes) [65]. No CSPs from the German cockroach have been previously reported. We identified a total of 12 CSP-coding gene fragments in the present study, with 8 CSPs being homologous to CSP7 and CSP18 from *T. castaneum*. The four CSPs with a long coding region identified in the study (Figure 4B) contained the typical four-cysteine signature and a common cysteine sequence motif of C_1_-X_6-8_-C_2_-X_16-21_-C_3_-X_2_-C_4_ of insect CSPs [66].

**Figure 5:**
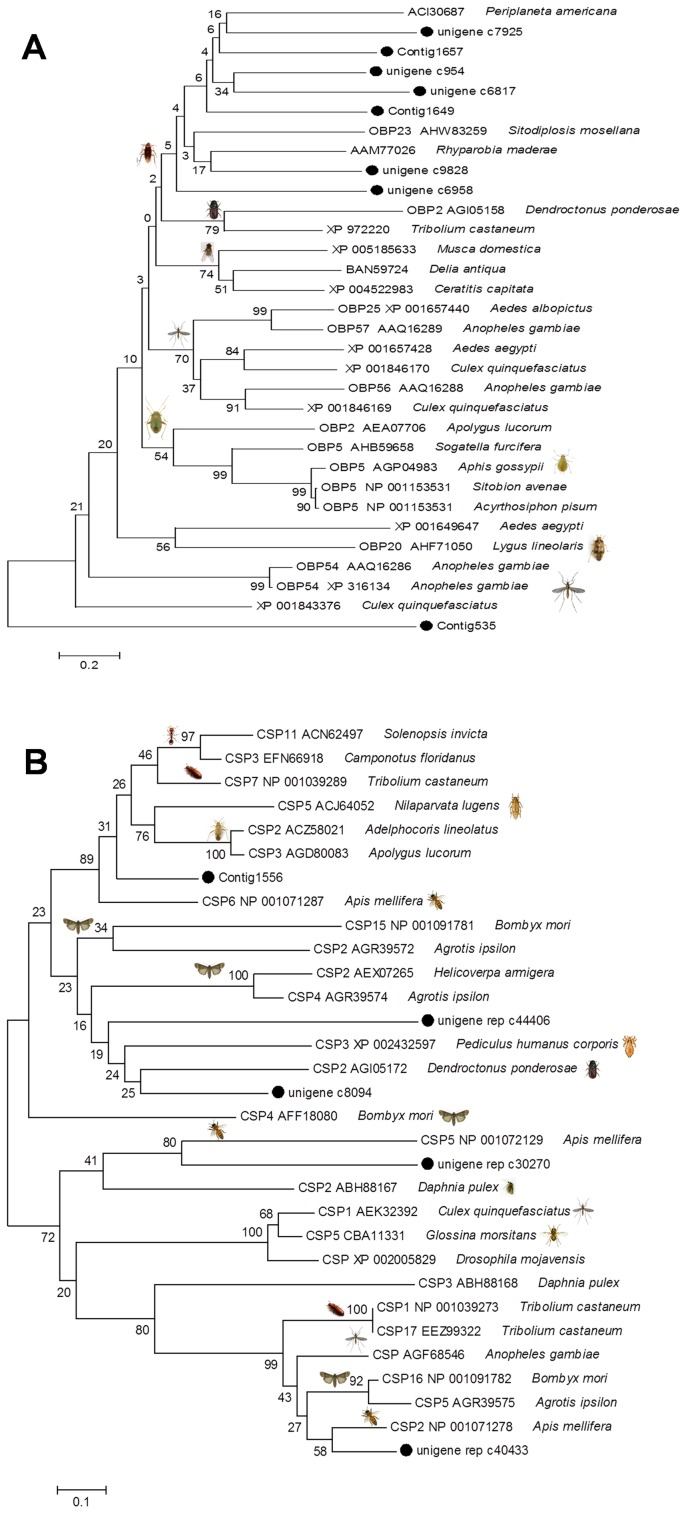
Phylogeny of the OBPs (A) and CSPs (B) from the German cockroach and their homologs. The unrooted consensus trees with 1000 bootstrap replicates are generated in MEGA6 [39] using the neighbor-joining method. The tree is drawn to scale, with branch lengths in the same units as those of the evolutionary distances used to infer the phylogenetic tree. All positions containing gaps and missing data are eliminated. GenBank accession numbers and species names of the sequences used here are shown in the phylogenetic trees. German cockroach OBPs and CSPs (marked by •) are in bolds.

In order to gain insight of the relationships between the annotated OBPs/CSPs and their counterparts from other insects, we carried out a phylogenetic analysis using putative amino acid sequences. The resultant tree constructed by the neighbor-joining method suggests that the 7 identified German cockroach OBPs were clustered into a group comprising related proteins of the other two cockroach species (*P. Americana* and *Rhyparobia Maderae*) and *Sitodiplosis mosellana* (orange wheat blossom midge), whereas the Contig 535 was dispersed at the bottom of the tree (Figure 5A). Consistent with their diverse functions, OBPs have a high sequence divergence (20%–30% of amino acid identity, see OBPs alignment in supporting information) [60], [67]. Insect OBPs are likely to be involved in broader physiological functions, not restricted to olfaction [68]. In contrast, CSPs from diverse insect species share high amino acid identities (about 50%–55%, Figure 5B), supporting the view that insect CSPs are highly conserved even across very distant species, and implying important roles they might play in insect physiology [69].

ORs are central to odorant detection in insects. Because of their high sequence variability, the most common method to identify OR-coding genes is to analyze genomic sequence databases [70]. Only 2 OR unigenes were annotated in this transcriptome, far fewer than those from other insects.

**Figure 6:**
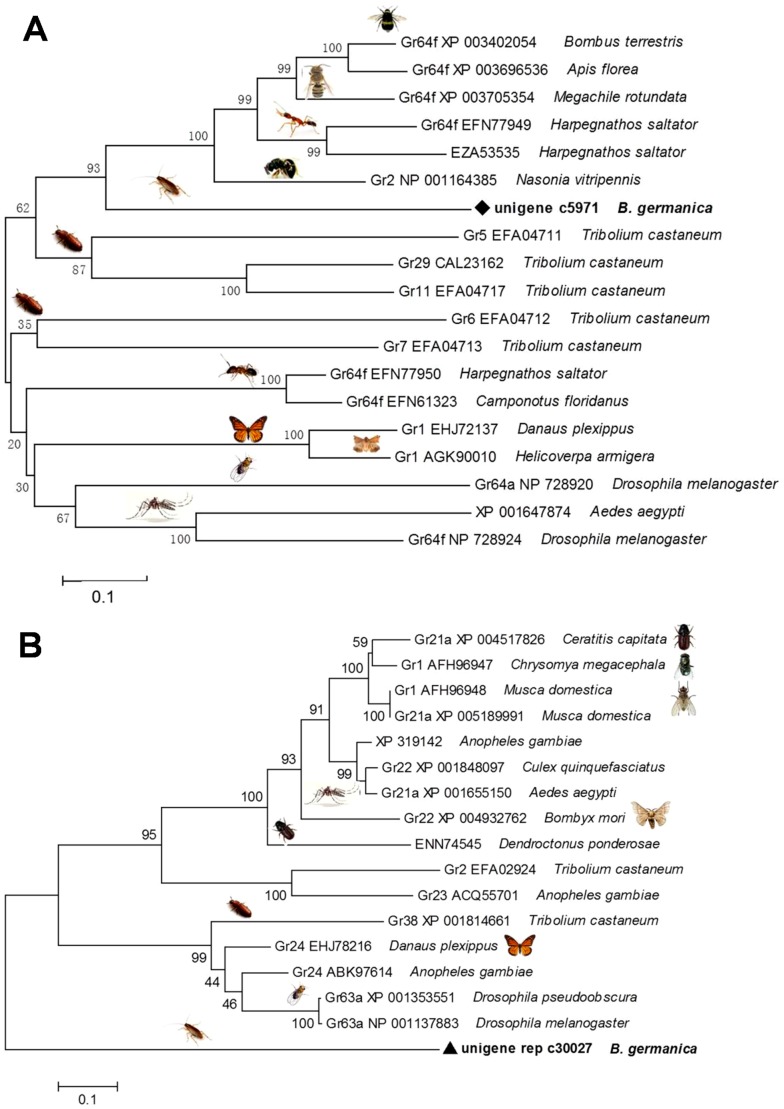
Phylogeny of two gustatory receptor genes from the German cockroach. Unigenes c5791 (A) and c rep c30027 (B) are highlighted in bolds and triangles (▴).

It has been reported that *Drosophila* contains a family of 60 gustatory receptor (GR) genes [71]. We identified 4 transcripts encoding homologous gustatory receptor in the cockroach. Two of them (unigene c5971 and unigene rep c30027) with a size of over 500 bp were exploited to analyze their phylogeny. As shown in Figure 6A, unigene c5971 showed homology to several other GRs from different species, such as Gr64f from *D. melanogaster*. Gr64f and other seven proteins (Gr5a, Gr61a and Gr64a–f) have been identified as sugar receptors (SRs) in *D. melanogaster* [72]. *Drosophila* sugar receptors function as multimers, and Gr64f is required broadly as a co-receptor for the detection of sugars [73]. We propose that c5971 gene identified in this study may also be involved in sugar perception of German cockroach. Unigene rep c30027 similarly showed close evolutionary distances with Gr63a and Gr21a (Figure 6B), which are co-expressed in CO_2_-responsive neurons and play an important role in the fruit fly food-seeking [74], suggesting the involvement of unigene rep c30027 in food seeking in the cockroach. Conservation of these GR sequences between fairly diverged insect species likely reflects indispensable gustatory sensitivities to a particular chemical or set of chemicals, a property that allows us to speculate their potential function.

## Conclusions

This study provides a new genetic data resource helpful for further comprehensive studies on the German cockroach. The information presented here will be useful to improve our understanding about the molecular mechanisms of cockroach immunity, insecticide resistance, chemoreception and gene regulation.

## Supporting Information

Figure S1 **Three GO sub-categories for German cockroach transcriptome.** (A) Biological process, (B) Cellular component and (C) Molecular function.
(TIF)

Table S1 **Bioassay of German cockroach susceptible (BJ-S) and resistant (DX-R) strains to different insecticide (KT_50_, min) using glass test method (WHO, 1963).**
(DOCX)

Table S2 **Summary of KEGG pathway types in the transcriptome of German cockroach.**
(DOCX)

File S1 **Sequences of CSP, GR, OBP, OR and top 20 abundant unigenes mentioned in the study.**
(ZIP)

## References

[1] Appel AG (1995) *Blattella* and related species. In: Rust MK, Owens JM, Reierson DA, editors. Understanding and Controlling the German Cockroach. New York: Oxford Press. pp. 1–19.

[2] Ross MH, Cochran DG (1975) The German cockroach, *Blattella germanica*. In: King RC, editor. Handbook of Genetics. New York: Plenum Press. pp. 35–62.

[3] Brenner RJ (1995) Economics and medical importance of German cockroaches. In: Rust MK, Owens JM, Reierson DA, editors. Understanding and Controlling the German Cockroach, New York: Oxford Press. pp. 77–92.

[4] Ramirez PJ (1989) The cockroach as a vector of pathogenic agents. Bol Oficina Sanit Panam 107: 41–53. 2529868

[5] Rosenstreich DL, Eggleston P, Kattan M, Baker D, Slavin RG, et al (1997) The role of cockroach allergy and exposure to cockroach allergen in causing morbidity among inner-city children with asthma. N Engl J Med 336: 1356–1363. 9134876 10.1056/NEJM199705083361904

[6] Gore JC, Schal C (2007) Cockroach Allergen Biology and Mitigation in the Indoor Environment. Annu Rev Entomol 52: 439–463. 17163801 10.1146/annurev.ento.52.110405.091313

[7] Irles P, Bellés X, Piulachs MD (2009) Identifying genes related to choriogenesis in insect panoistic ovaries by Suppression Subtractive Hybridization. BMC Genomics 10: 206. 19405973 10.1186/1471-2164-10-206 PMC2683872

[8] Chung HS, Yu TH, Kim BJ, Kim SM, Kim JY, et al (2005) Expressed sequence tags analysis of *Blattella germanica* . Korean J Parasitol 43: 149–156. 16340304 10.3347/kjp.2005.43.4.149 PMC2712019

[9] Feyereisen R (2012) Insect CYP Genes and P450 Enzymes. In: Gilbert LI editor. Insect Molecular Biology and Biochemistry. London: Academic Press. pp. 236–345.

[10] Shendure J, Ji H (2008) Next-generation DNA sequencing. Nat Biotechnol 26: 1135–1145. 18846087 10.1038/nbt1486

[11] Schuster SC (2008) Next-generation sequencing transforms today's biology. Nat Methods 5: 16–18. 18165802 10.1038/nmeth1156

[12] Margulies M, Egholm M, Altman WE, Attiya S, Bader JS, et al (2005) Genome sequencing in microfabricated high-density picolitre reactors. Nature 437: 376–380. 16056220 10.1038/nature03959 PMC1464427

[13] Vera JC, Wheat CW, Fescemyer HW, Frilander MJ, Crawford DL, et al (2008) Rapid transcriptome characterization for a nonmodel organism using 454 pyrosequencing. Mol Ecol 17: 1636–1647. 18266620 10.1111/j.1365-294X.2008.03666.x

[14] Droege M, Hill B (2008) The Genome Sequencer FLX System-longer reads, more applications, straight forward bioinformatics and more complete data sets. J Biotechnol 136: 3–10. 18616967 10.1016/j.jbiotec.2008.03.021

[15] Bai X, Mamidala P, Rajarapu SP, Jones SC, Mittapalli O (2011) Transcriptomics of the bed bug (*Cimex lectularius*). PLoS One 6: e16336. 21283830 10.1371/journal.pone.0016336 PMC3023805

[16] Adelman ZN, Kilcullen KA, Koganemaru R, Anderson MA, Anderson TD, et al (2011) Deep sequencing of pyrethroid-resistant bed bugs reveals multiple mechanisms of resistance within a single population. PLoS One 6: e26228. 22039447 10.1371/journal.pone.0026228 PMC3198472

[17] Gregory R, Darby AC, Irving H, Coulibaly MB, Hughes M, et al (2011) A *de novo* expression profiling of *Anopheles funestus*, malaria vector in Africa, using 454 pyrosequencing. PLoS One 6: e17418. 21364769 10.1371/journal.pone.0017418 PMC3045460

[18] Price DP, Nagarajan V, Churbanov A, Houde P, Milligan B, et al (2011) The fat body transcriptomes of the yellow fever mosquito *Aedes aegypti*, pre- and post- blood meal. PLoS One 6: e22573. 21818341 10.1371/journal.pone.0022573 PMC3144915

[19] Jaworski DC, Zou Z, Bowen CJ, Wasala NB, Madden R, et al (2010) Pyrosequencing and characterization of immune response genes from the American dog tick, *Dermacentor variabilis* (L.). Insect Mol Biol 19: 617–630. 20698900 10.1111/j.1365-2583.2010.01037.x PMC9327058

[20] Liu F, Tang T, Sun L, Jose Priya TA (2012) Transcriptomic analysis of the housefly (*Musca domestica*) larva using massively parallel pyrosequencing. Mol Biol Rep 39: 1927–1934. 21643958 10.1007/s11033-011-0939-3

[21] Hahn DA, Ragland GJ, Shoemaker DD, Denlinger DL (2009) Gene discovery using massively parallel pyrosequencing to develop ESTs for the flesh fly *Sarcophaga crassipalpis* . BMC Genomics 10: 234. 19454017 10.1186/1471-2164-10-234 PMC2700817

[22] Carvalho RA, Azeredo-Espin AM, Torres TT (2010) Deep sequencing of New World screw-worm transcripts to discover genes involved in insecticide resistance. BMC Genomics 11: 695. 21143848 10.1186/1471-2164-11-695 PMC3022914

[23] Nirmala X, Schetelig MF, Yu F, Handler AM (2013) An EST database of the Caribbean fruit fly, *Anastrepha suspensa* (Diptera: Tephritidae). Gene 517: 212–217. 23296060 10.1016/j.gene.2012.12.012

[24] Peng X, Zha W, He R, Lu T, Zhu L, et al (2011) Pyrosequencing the midgut transcriptome of the brown planthopper, *Nilaparvata lugens* . Insect Mol Biol 20: 745–762. 21919985 10.1111/j.1365-2583.2011.01104.x

[25] Pauchet Y, Wilkinson P, Vogel H, Nelson DR, Reynolds SE, et al (2010) Pyrosequencing the *Manduca sexta* larval midgut transcriptome: messages for digestion, detoxification and defence. Insect Mol Biol 19: 61–75. 19909380 10.1111/j.1365-2583.2009.00936.x

[26] de Moura TR, Oliveira F, Carneiro MW, Miranda JC, Clarêncio J, et al (2013) Functional transcriptomics of wild-caught *Lutzomyia intermedia* salivary glands: identification of a protective salivary protein against *Leishmania braziliensis* infection. PLoS Negl Trop Dis 7: e2242. 23717705 10.1371/journal.pntd.0002242 PMC3662654

[27] Karim S, Singh P, Ribeiro JM (2011) A deep insight into the sialotranscriptome of the gulf coast tick, *Amblyomma maculatum* . PLoS One 6: e28525. 22216098 10.1371/journal.pone.0028525 PMC3244413

[28] Ribeiro JM, Chagas AC, Pham VM, Lounibos LP, Calvo E (2014) An insight into the sialome of the frog biting fly, *Corethrella appendiculata* . Insect Biochem Mol Biol 44: 23–32. 10.1016/j.ibmb.2013.10.006 PMC4035455 24514880

[29] Ribeiro JM, Genta FA, Sorgine MH, Logullo R, Mesquita RD, et al (2014) An insight into the transcriptome of the digestive tract of the bloodsucking bug, *Rhodnius prolixus* . PLoS Negl Trop Dis 8: e2594. 24416461 10.1371/journal.pntd.0002594 PMC3886914

[30] Cristino AS, Tanaka ED, Rubio M, Piulachs MD, Belles X (2011) Deep sequencing of organ- and stage-specific microRNAs in the evolutionarily basal insect *Blattella germanica* (*L.*) (Dictyoptera, Blattellidae). PLoS One 6: e19350. 21552535 10.1371/journal.pone.0019350 PMC3084283

[31] Lassmann T, Hayashizaki Y, Daub CO (2009) TagDust: a program to eliminate artifacts from next generation sequencing data. Bioinformatics 25: 2839–2840. 19737799 10.1093/bioinformatics/btp527 PMC2781754

[32] Chen YA, Lin CC, Wang CD, Wu HB, Hwang PI (2007) An optimized procedure greatly improves EST vector contamination removal. BMC Genomics 8: 416. 17997864 10.1186/1471-2164-8-416 PMC2194723

[33] Boeckmann B, Bairoch A, Apweiler R, Blatter MC, Estreicher A, et al (2003) The SWISS-PROT protein knowledgebase and its supplement TrEMBL in 2003. Nucleic Acids Res 31: 365–370. 12520024 10.1093/nar/gkg095 PMC165542

[34] Tatusov RL, Fedorova ND, Jackson JD, Jacobs AR, Kiryutin B, et al (2003) The COG database: an updated version includes eukaryotes. BMC Bioinformatics 4: 41. 12969510 10.1186/1471-2105-4-41 PMC222959

[35] Kanehisa M, Araki M, Goto S, Hattori M, Hirakawa M, et al (2008) KEGG for linking genomes to life and the environment. Nucleic Acids Res 36: D480–4. 18077471 10.1093/nar/gkm882 PMC2238879

[36] Conesa A, Götz S (2008) Blast2GO: a comprehensive suite for functional analysis in plant genomics. Int J Plant Genomics 2008: 619832. 18483572 10.1155/2008/619832 PMC2375974

[37] Gotz S, Garcia-Gomez JM, Terol J, Williams TD, Nagaraj SH, et al (2008) High-throughput functional annotation and data mining with the Blast2GO suite. Nucleic Acids Res 36: 3420–3435. 18445632 10.1093/nar/gkn176 PMC2425479

[38] Ye J, Fang L, Zheng H, Zhang Y, Chen J, et al (2006) WEGO: a web tool for plotting GO annotations. Nucleic Acids Res 34: W293–7. 16845012 10.1093/nar/gkl031 PMC1538768

[39] Tamura K, Stecher G, Peterson D, Filipski A, Kumar S (2013) MEGA6: Molecular Evolutionary Genetics Analysis version 6.0. Mol Biol Evol 30: 2725–2729. 24132122 10.1093/molbev/mst197 PMC3840312

[40] Wang XW, Luan JB, Li JM, Bao YY, Zhang CX, et al (2010) *De novo* characterization of a whitefly transcriptome and analysis of its gene expression during development. BMC Genomics 11: 400. 20573269 10.1186/1471-2164-11-400 PMC2898760

[41] Niu JZ, Dou W, Ding TB, Shen GM, Zhang K, et al (2012) Transcriptome analysis of the citrus red mite, *Panonychus citri*, and its gene expression by exposure to insecticide/acaricide. Insect Mol Biol 21: 422–436. 22676046 10.1111/j.1365-2583.2012.01148.x

[42] Garbutt JS, Belles X, Richards EH, Reynolds SE (2013) Persistence of double-stranded RNA in insect hemolymph as a potential determiner of RNA interference success: evidence from *Manduca sexta* and *Blattella germanica* . J Insect Physiol 59: 171–178. 22664137 10.1016/j.jinsphys.2012.05.013

[43] Huvenne H, Smagghe G (2010) Mechanisms of dsRNA uptake in insects and potential of RNAi for pest control: a review. J Insect Physiol 56: 227–235. 19837076 10.1016/j.jinsphys.2009.10.004

[44] Ulvila J, Parikka M, Kleino A, Sormunen R, Ezekowitz RA, et al (2006) Double-stranded RNA is internalized by scavenger receptor-mediated endocytosis in *Drosophila* S2 cells. J Biol Chem 281: 14370–14375. 16531407 10.1074/jbc.M513868200

[45] Shen GM, Dou W, Huang Y, Jiang XZ, Smagghe G, et al (2013) *In silico* cloning and annotation of genes involved in the digestion, detoxification and RNA interference mechanism in the midgut of *Bactrocera dorsalis* [Hendel (Diptera: Tephritidae)]. Insect Mol Biol 22: 354–365. 23577657 10.1111/imb.12026

[46] Jiang F, Ye X, Liu X, Fincher L, McKearin D, et al (2005) Dicer-1 and R3D1-L catalyze microRNA maturation in *Drosophila* . Genes Dev 19: 1674–1679. 15985611 10.1101/gad.1334005 PMC1176004

[47] Liu Q, Rand TA, Kalidas S, Du F, Kim HE, et al (2003) R2D2, a bridge between the initiation and effector steps of the *Drosophila* RNAi pathway. Science 301: 1921–1925. 14512631 10.1126/science.1088710

[48] Gomez-Orte E, Belles X (2009) MicroRNA-dependent metamorphosis in hemimetabolan insects. Proc Natl Acad Sci USA 106: 21678–21682. 19966227 10.1073/pnas.0907391106 PMC2799836

[49] Matranga C, Tomari Y, Shin C, Bartel DP, Zamore PD (2005) Passenger-strand cleavage facilitates assembly of siRNA into Ago2-containing RNAi enzyme complexes. Cell 123: 607–620. 16271386 10.1016/j.cell.2005.08.044

[50] Wei Y, Appel AG, Moar WJ, Liu N (2001) Pyrethroid resistance and cross-resistance in the German cockroach, *Blattella germanica* (L). Pest Manag Sci 57: 1055–1059. 11721523 10.1002/ps.383

[51] Valles SM (1998) Toxicological and biochemical studies with field populations of the German cockroach, *Blattella germanica* . Pestic Biochem Physiol 62: 190–200.

[52] Liu Z, Valles SM, Dong K (2000) Novel point mutations in the German cockroach para sodium channel gene are associated with knockdown resistance (kdr) to pyrethroid insecticides. Insect Biochem Mol Biol 30: 991–997. 10899465 10.1016/s0965-1748(00)00074-6 PMC3049294

[53] Wada-Katsumata A, Silverman J, Schal C (2013) Changes in taste neurons support the emergence of an adaptive behavior in cockroaches. Science 340: 972–975. 23704571 10.1126/science.1234854

[54] Oakeshott JG, Johnson RM, Berenbaum MR, Ranson H, Cristino AS, et al (2010) Metabolic enzymes associated with xenobiotic and chemosensory responses in *Nasonia vitripennis* . Insect Mol Biol 19 Suppl 1147–163. 20167025 10.1111/j.1365-2583.2009.00961.x

[55] Lee S, Siddiqui R, Khan NA (2012) Animals living in polluted environments are potential source of antimicrobials against infectious agents. Pathog Glob Health 106: 218–223. 23265422 10.1179/2047773212Y.0000000033 PMC4001588

[56] Hoffmann JA, Reichhart JM (2002) *Drosophila* innate immunity: an evolutionary perspective. Nature Immunol 3: 121–126. 11812988 10.1038/ni0202-121

[57] Kim T, Kim YJ (2005) Overview of innate immunity in *Drosophila* . J Biochem Mol Biol 38: 121–127. 15826489 10.5483/bmbrep.2005.38.2.121

[58] Hekmat-Scafe DS, Scafe CR, McKinney AJ, Tanouye MA (2002) Genome-wide analysis of the odorant-binding protein gene family in *Drosophila melanogaster* . Genome Res 12: 1357–1369. 12213773 10.1101/gr.239402 PMC186648

[59] Gong DP, Zhang HJ, Zhao P, Xia QY, Xiang ZH (2009) The odorant binding protein gene family from the genome of silkworm, *Bombyx mori* . BMC Genomics 10: 332. 19624863 10.1186/1471-2164-10-332 PMC2722677

[60] Manoharan M, Ng Fuk Chong M, Vaïtinadapoulé A, Frumence E, Sowdhamini R, et al (2013) Comparative genomics of odorant binding proteins in *Anopheles gambiae*, *Aedes aegypti*, and *Culex quinquefasciatus* . Genome Biol Evol 5: 163–180. 23292137 10.1093/gbe/evs131 PMC3595023

[61] Gotzek D, Robertson HM, Wurm Y, Shoemaker D (2011) Odorant binding proteins of the red imported fire ant, *Solenopsis invicta*: an example of the problems facing the analysis of widely divergent proteins. PLoS One 6: e16289. 21305009 10.1371/journal.pone.0016289 PMC3031547

[62] Kirkness EF, Haas BJ, Sun W, Braig HR, Perotti MA, et al (2010) Genome sequences of the human body louse and its primary endosymbiont provide insights into the permanent parasitic lifestyle. Proc Natl Acad Sci USA 107: 12168–12173. 20566863 10.1073/pnas.1003379107 PMC2901460

[63] Kulmuni J, Havukainen H (2013) Insights into the evolution of the CSP gene family through the integration of evolutionary analysis and comparative protein modeling. PLoS One 8: e63688. 23723994 10.1371/journal.pone.0063688 PMC3665776

[64] Wanner KW, Willis LG, Theilmann DA, Isman MB, Feng Q, et al (2004) Analysis of the insect os-d-like gene family. J Chem Ecol 30: 889–911. 15274438 10.1023/b:joec.0000028457.51147.d4

[65] Vieira FG, Rozas J (2011) Comparative Genomics of the Odorant-Binding and Chemosensory Protein Gene Families across the Arthropoda: Origin and Evolutionary History of the Chemosensory System. Genome Biology and Evolution 3: 476–490. 21527792 10.1093/gbe/evr033 PMC3134979

[66] Zhou JJ, Kan Y, Antoniw J, Pickett JA, Field LM (2006) Genome and EST analyses and expression of a gene family with putative functions in insect chemoreception. Chem Senses 31: 453–465. 16581978 10.1093/chemse/bjj050

[67] Gu SH, Wang SY, Zhang XY, Ji P, Liu JT, et al (2012) Functional characterizations of chemosensory proteins of the alfalfa plant bug *Adelphocoris lineolatus* indicate their involvement in host recognition. PLoS One 7: e42871. 22900060 10.1371/journal.pone.0042871 PMC3416781

[68] Hekmat-Scafe DS, Scafe CR, McKinney AJ, Tanouye MA (2002) Genome-wide analysis of the odorant-binding protein gene family in *Drosophila melanogaster* . Genome Res 12: 1357–1369. 12213773 10.1101/gr.239402 PMC186648

[69] Pelosi P, Zhou JJ, Ban LP, Calvello M (2006) Soluble proteins in insect chemical communication. Cell Mol Life Sci 63: 1658–1676. 16786224 10.1007/s00018-005-5607-0 PMC11136032

[70] Grosse-Wilde E, Kuebler LS, Bucks S, Vogel H, Wicher D, et al (2011) Antennal transcriptome of *Manduca sexta* . Proc Natl Acad Sci USA 108: 7449–7454. 21498690 10.1073/pnas.1017963108 PMC3088587

[71] Robertson HM, Warr CG, Carlson JR (2003) Molecular evolution of the insect chemoreceptor gene superfamily in *Drosophila melanogaster* . Proc Natl Acad Sci USA 100: 14537–14542. 14608037 10.1073/pnas.2335847100 PMC304115

[72] Kent LB, Robertson HM (2009) Evolution of the sugar receptors in insects. BMC Evol Biol 9: 41. 19226470 10.1186/1471-2148-9-41 PMC2667405

[73] Jiao Y, Moon SJ, Wang X, Ren Q, Montell C (2008) Gr64f is required in combination with other gustatory receptors for sugar detection in *Drosophila* . Curr Biol 18: 1797–7801. 19026541 10.1016/j.cub.2008.10.009 PMC2676565

[74] Turner SL, Ray A (2009) Modification of CO_2_ avoidance behaviour in *Drosophila* by inhibitory odorants. Nature 461: 277–281. 19710651 10.1038/nature08295

