# Supplementary figures and images for: De Novo Transcriptome of the Hemimetabolous German Cockroach (Blattella germanica)
Source: PLoS One. 2014 Sep 29;9(9):e106932. doi: 10.1371/journal.pone.0106932 (PMC4180286; doi:10.1371/journal.pone.0106932)

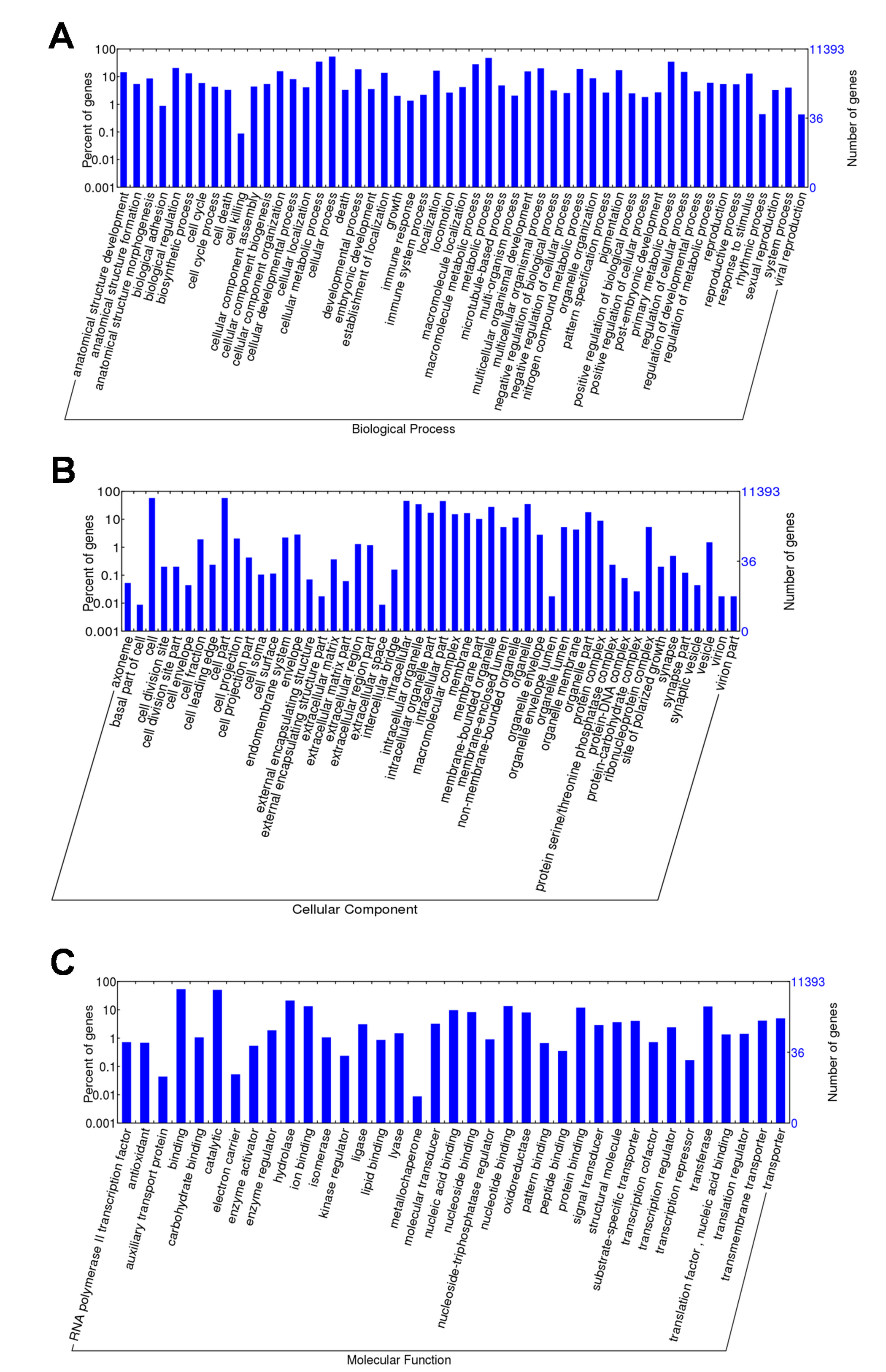

Supplement: Figure S1 — Three GO sub-categories for German cockroach transcriptome. (A) Biological process, (B) Cellular component and (C) Molecular function. (TIF) [file pone.0106932.s001.tif]
