# Supplementary material for: De Novo Transcriptome of the Hemimetabolous German Cockroach (Blattella germanica)
Source: PLoS One. 2014 Sep 29;9(9):e106932. doi: 10.1371/journal.pone.0106932 (PMC4180286; doi:10.1371/journal.pone.0106932)
Supplement: Table S1 — Bioassay of German cockroach susceptible (BJ-S) and resistant (DX-R) strains to different insecticide (KT50, min) using glass test method (WHO, 1963). (DOCX) [file pone.0106932.s002.docx]

**Table S1 Bioassay of German cockroach susceptible (BJ-S) and resistant (DX-R) strains to different insecticide (KT_50_, min) using glass test method (WHO, 1963)**

| **Strain** | **Insecticide** | **n** | **KT_50_ (min)**  **(95% CI)** | **RR*** |
| --- | --- | --- | --- | --- |
| **BJ-S** | Propoxur | 90 | 11.36 (11.00-11.73) | - |
|  | β-cypermethrin | 90 | 2.37 (2.27-2.46) | - |
|  | Acephate | 90 | 40.46 (37.53-43.16) | - |
| **DX-R** | Propoxur | 100 | 46.15 (38.50-55.19) | 4.06 |
|  | β-cypermethrin | 110 | 11.25 (8.70-13.92 ) | 4.75 |
|  | Acephate | 100 | 99.27 (72.06-124.58) | 2.45 |

* RR, resistance ratio at the 50% knockdown time (KT_50_ DX-R /KT_50_ BJ-S).
