# Supplementary material for: De Novo Transcriptome of the Hemimetabolous German Cockroach (Blattella germanica)
Source: PLoS One. 2014 Sep 29;9(9):e106932. doi: 10.1371/journal.pone.0106932 (PMC4180286; doi:10.1371/journal.pone.0106932)
Supplement: Table S2 — Summary of KEGG pathway types in the transcriptome of German cockroach. (DOCX) [file pone.0106932.s003.docx]

**Table S2. Summary of KEGG pathway types in the transcriptome of German cockroach**

| KEGG pathway tyes | Frequency |
| --- | --- |
| Vitamin digestion and absorption | 97 |
| mRNA surveillance pathway | 171 |
| Insect hormone biosynthesis | 75 |
| Apoptosis | 110 |
| Terpenoid backbone biosynthesis | 66 |
| Sulfur metabolism | 58 |
| Retinol metabolism | 519 |
| Arginine and proline metabolism | 230 |
| ABC transporters | 233 |
| Glycerolipid metabolism | 344 |
| Pentose phosphate pathway | 219 |
| SNARE interactions in vesicular transport | 81 |
| Cyanoamino acid metabolism | 133 |
| Complement and coagulation cascades | 132 |
| Glycosaminoglycan degradation | 103 |
| Steroid hormone biosynthesis | 446 |
| RNA transport | 502 |
| RIG-I-like receptor signaling pathway | 69 |
| Glycine, serine and threonine metabolism | 285 |
| Cysteine and methionine metabolism | 140 |
| Adipocytokine signaling pathway | 137 |
| RNA degradation | 335 |
| Cytokine-cytokine receptor interaction | 82 |
| Phenylpropanoid biosynthesis | 156 |
| Nitrogen metabolism | 110 |
| Olfactory transduction | 181 |
| Glycosphingolipid biosynthesis - ganglio series | 28 |
| Aminobenzoate degradation | 686 |
| Tropane, piperidine and pyridine alkaloid biosynthesis | 200 |
| Glycosphingolipid biosynthesis - globo series | 52 |
| Pyruvate metabolism | 310 |
| Linoleic acid metabolism | 376 |
| beta-Alanine metabolism | 137 |
| Purine metabolism | 991 |
| Selenocompound metabolism | 76 |
| Basal transcription factors | 177 |
| Styrene degradation | 25 |
| Proteasome | 219 |
| Proximal tubule bicarbonate reclamation | 73 |
| Sphingolipid metabolism | 123 |
| Phototransduction | 93 |
| Glycosaminoglycan biosynthesis - chondroitin sulfate | 70 |
| Nicotinate and nicotinamide metabolism | 73 |
| Flavone and flavonol biosynthesis | 23 |
| Stilbenoid, diarylheptanoid and gingerol biosynthesis | 311 |
| Sulfur relay system | 21 |
| Valine, leucine and isoleucine degradation | 195 |
| Endocytosis | 457 |
| Lysine degradation | 417 |
| Vasopressin-regulated water reabsorption | 135 |
| Pantothenate and CoA biosynthesis | 86 |
| MAPK signaling pathway | 433 |
| Synthesis and degradation of ketone bodies | 44 |
| Ribosome biogenesis in eukaryotes | 107 |
| Bisphenol degradation | 428 |
| One carbon pool by folate | 66 |
| Toll-like receptor signaling pathway | 149 |
| Other types of O-glycan biosynthesis | 190 |
| Taste transduction | 16 |
| Pentose and glucuronate interconversions | 417 |
| Cytosolic DNA-sensing pathway | 62 |
| TGF-beta signaling pathway | 124 |
| Aldosterone-regulated sodium reabsorption | 84 |
| Polycyclic aromatic hydrocarbon degradation | 373 |
| Phenylalanine metabolism | 116 |
| Phenylalanine, tyrosine and tryptophan biosynthesis | 35 |
| Glycosphingolipid biosynthesis - lacto and neolacto series | 13 |
| Cell cycle - Caulobacter | 16 |
| Vitamin B6 metabolism | 15 |
| Galactose metabolism | 270 |
| Tryptophan metabolism | 219 |
| Limonene and pinene degradation | 461 |
| Carbon fixation in photosynthetic organisms | 136 |
| Notch signaling pathway | 115 |
| Propanoate metabolism | 151 |
| Fatty acid biosynthesis | 69 |
| Ubiquitin mediated proteolysis | 553 |
| NOD-like receptor signaling pathway | 90 |
| Biosynthesis of secondary metabolites | 2182 |
| Wnt signaling pathway | 400 |
| Alanine, aspartate and glutamate metabolism | 173 |
| Plant-pathogen interaction | 229 |
| Lipoic acid metabolism | 12 |
| Asthma | 10 |
| Fatty acid metabolism | 240 |
| Spliceosome | 837 |
| Metabolic pathways | 5904 |
| Ascorbate and aldarate metabolism | 340 |
| Tyrosine metabolism | 399 |
| Caffeine metabolism | 28 |
| Phosphonate and phosphinate metabolism | 27 |
| Atrazine degradation | 9 |
| Thiamine metabolism | 10 |
| Nucleotide excision repair | 182 |
| Metabolism of xenobiotics by cytochrome P450 | 606 |
| Primary bile acid biosynthesis | 26 |
| RNA polymerase | 494 |
| Biosynthesis of unsaturated fatty acids | 234 |
| Fructose and mannose metabolism | 260 |
| Histidine metabolism | 134 |
| Calcium signaling pathway | 388 |
| Cell adhesion molecules (CAMs) | 180 |
| Folate biosynthesis | 43 |
| Pyrimidine metabolism | 746 |
| Taurine and hypotaurine metabolism | 21 |
| Peroxisome | 403 |
| Glycosaminoglycan biosynthesis - keratan sulfate | 7 |
| Intestinal immune network for IgA production | 7 |
| Fat digestion and absorption | 155 |
| Two-component system | 72 |
| Arachidonic acid metabolism | 168 |
| Gap junction | 179 |
| Benzoate degradation | 140 |
| Caprolactam degradation | 88 |
| Fatty acid elongation in mitochondria | 51 |
| Glyoxylate and dicarboxylate metabolism | 106 |
| Zeatin biosynthesis | 6 |
| Peptidoglycan biosynthesis | 6 |
| Photosynthesis | 6 |
| Carotenoid biosynthesis | 6 |
| Chloroalkane and chloroalkene degradation | 136 |
| Mucin type O-Glycan biosynthesis | 35 |
| Cell cycle | 377 |
| Glycerophospholipid metabolism | 193 |
| Mineral absorption | 115 |
| Plant hormone signal transduction | 48 |
| Methane metabolism | 168 |
| Isoquinoline alkaloid biosynthesis | 151 |
| Protein export | 82 |
| Ribosome | 637 |
| Ubiquinone and other terpenoid-quinone biosynthesis | 59 |
| Natural killer cell mediated cytotoxicity | 114 |
| D-Glutamine and D-glutamate metabolism | 3 |
| D-Alanine metabolism | 4 |
| Lysine biosynthesis | 28 |
| Protein processing in endoplasmic reticulum | 670 |
| Biosynthesis of siderophore group nonribosomal peptides | 14 |
| Glycolysis / Gluconeogenesis | 349 |
| Long-term potentiation | 209 |
| Amino sugar and nucleotide sugar metabolism | 496 |
| ErbB signaling pathway | 139 |
| Indole alkaloid biosynthesis | 13 |
| Citrate cycle (TCA cycle) | 242 |
| Geraniol degradation | 23 |
| Ethylbenzene degradation | 82 |
| Carbohydrate digestion and absorption | 196 |
| Butanoate metabolism | 200 |
| Glutathione metabolism | 335 |
| Riboflavin metabolism | 142 |
| N-Glycan biosynthesis | 107 |
| Naphthalene degradation | 127 |
| Aminoacyl-tRNA biosynthesis | 332 |
| Xylene degradation | 2 |
| Allograft rejection | 2 |
| Chlorocyclohexane and chlorobenzene degradation | 2 |
| DDT degradation | 2 |
| Dioxin degradation | 2 |
| Phosphatidylinositol signaling system | 283 |
| Base excision repair | 159 |
| GnRH signaling pathway | 191 |
| Endocrine and other factor-regulated calcium reabsorption | 122 |
| Glycosylphosphatidylinositol(GPI)-anchor biosynthesis | 74 |
| Starch and sucrose metabolism | 646 |
| Betalain biosynthesis | 116 |
| Butirosin and neomycin biosynthesis | 20 |
| Porphyrin and chlorophyll metabolism | 248 |
| Valine, leucine and isoleucine biosynthesis | 208 |
| Long-term depression | 105 |
| Ether lipid metabolism | 73 |
| Mismatch repair | 96 |
| DNA replication | 176 |
| Inositol phosphate metabolism | 229 |
| Protein digestion and absorption | 378 |
| Homologous recombination | 92 |
| alpha-Linolenic acid metabolism | 48 |
| Focal adhesion | 871 |
| Fc epsilon RI signaling pathway | 87 |
| Steroid biosynthesis | 100 |
| Biotin metabolism | 23 |
| Glycosaminoglycan biosynthesis-heparan sulfate | 12 |
| Non-homologous end-joining | 31 |
| Oxidative phosphorylation | 525 |
| Various types of N-glycan biosynthesis | 70 |
| Tight junction | 755 |
| D-Arginine and D-ornithine metabolism | 3 |
| Toluene degradation | 3 |
| Phagosome | 919 |
| Other glycan degradation | 116 |
| Adherens junction | 381 |
| Lysosome | 750 |
